# Artemether Activation of AMPK/GSK3*β*(ser9)/Nrf2 Signaling Confers Neuroprotection towards *β-*Amyloid-Induced Neurotoxicity in 3xTg Alzheimer's Mouse Model

**DOI:** 10.1155/2019/1862437

**Published:** 2019-11-21

**Authors:** Shuai Li, Xia Zhao, Philip Lazarovici, Wenhua Zheng

**Affiliations:** ^1^Centre for Reproduction, Development & Aging, Faculty of Health Sciences, University of Macau, Taipa, Macau SAR, China; ^2^Institute of Translational Medicine, Faculty of Health Sciences, University of Macau, Taipa, Macau SAR, China; ^3^School of Pharmacy Institute for Drug Research, Faculty of Medicine, The Hebrew University of Jerusalem, Jerusalem 91120, Israel

## Abstract

Alzheimer's disease is a severe neurodegenerative disease. Multiple factors involving neurofibrillary tangles and amyloid-*β* plaques lead to the progression of the AD, generated by aggregated hyperphosphorylated Tau protein. Inflammation, mitochondrial dysfunction, and oxidative stress play a significant role in the progression of AD. It has been therefore suggested that the multifactorial nature of AD pathogenesis requires the design of antioxidant drugs with a broad spectrum of neuroprotective activities. For this reason, the use of natural products, characterized by multiple pharmacological properties is advantageous as AD-modifying drugs over the single-targeted chemicals. Artemether, a peroxide sesquiterpenoid lipid-soluble compound, has been used in the clinic as an antimalarial drug. Also, it exhibits potent anti-inflammatory and antioxidant activities. Here, we report the neuroprotective effects of Artemether towards A*β*-induced neurotoxicity in neuronal cell cultures. A temporal correlation was found between Artemether neuroprotection towards A*β*-induced neurotoxicity and AMPK/GSK3*β* phosphorylation activity and increased expression of the activated Nrf2 signaling pathway. In 3xTg-AD mice, Artemether attenuated learning and memory deficits, inhibited cortical neuronal apoptosis and glial activation, inhibited oxidative stress through decrease of lipid peroxidation and increased expression of SOD, and reduced A*β* deposition and tau protein phosphorylation. Moreover, in 3xTg-AD mice, Artemether induced phosphorylation of the AMPK/GSK3*β* pathway which activated Nrf2, increasing the level of antioxidant protein HO-1. These activities probably produced the antioxidant and anti-inflammatory effects responsible for the neuroprotective effects of Artemether in the 3xTg-AD mouse model. These findings propose Artemether as a new drug for the treatment of AD disease.

## 1. Introduction

Alzheimer's disease (AD) is the main cause of dementia in the elderly and the most common form of neurodegenerative diseases, characterized by the neurofibrillary tangles, deposition of amyloid plaques, followed by brain's neuronal loss [1]. With the significant increase in morbidity, AD is becoming a severe burden and has been identified as a priority in public health by the WHO [2]. Oxidative stress, inflammation, and elevated cholesterol levels are mechanisms that have been related to Alzheimer's disease [3]. *β*-amyloid's aggregates are thought to be the main component of senile plaques which have been found in the brain of AD patients leading to a high diversity of neurotoxic mechanisms [4] such as necrotic and apoptotic cell death [5], synaptic dysfunction [6], and memory deficits [7]. The conventional drug therapy of AD employs inhibitors of the enzyme acetylcholinesterase (e.g., donepezil, rivastigmine) thereby increasing levels of acetylcholine and glutamatergic antagonists (e.g., memantine) that protect the brain tissue against glutamate-mediated excitotoxicity. These drugs are ineffective in modifying the disease progression and more symptomatic. Since pathological, mechanism-oriented drug explorations have been less intensively investigated, there is an unmet clinical need to develop novel approaches towards AD therapy.

*β*-amyloid (A*β*) is produced by the subsequent cleavage of the amyloid precursor protein (APP) by *β*-site APP lyase 1 (BACE1) and *γ*-secretase, representing a major component of extracellular plaques [8]. The accumulation of A*β* in the brain caused an increase of intracellular reactive oxygen species (ROS) and mitochondrial oxidative stress and impaired calcium homeostasis, glucose regulation, and energy metabolism, ultimately leading to neuronal cell death by necrotic and apoptotic pathways [9, 10]. Cumulative studies show that oxidative stress plays a major role in the occurrence and development of AD [10, 11]. Hence, targeting oxidative stress using antioxidant drugs may offer new hope to patients suffering from this devastating disease [12]. There are many natural or plant products that have been used to ameliorate the symptoms and complications of AD [13]. Considering the multifactorial nature of AD pathogenesis, the search for an antioxidant drug with a broad spectrum of additional neuroprotective activities is recommended.

Artemisinin was originally isolated from the qinghao by Prof. Youyou Tu. The discovery of artemisinin has saved many malaria patients' lives. Over the past several decades, more than two hundred million malaria patients have received artemisinin or artemisinin combination therapies. In October 2015, Prof. Youyou Tu won the Nobel Prize in Physiology or Medicine. She became the first Chinese to receive the Nobel Prize in science. Artemisinin and its derivatives are among the most preferred first-line, effective, and safe therapies for malaria [14]. The related compounds such as dihydroartemisinin, artemisinic acid, artesunate, and Artemether are all derivatives of artemisinin. These drugs show a wide range of effects, such as antioxidant, anti-inflammatory, antimicrobial, antitumor, immunomodulatory, and neuroprotective effects [15–17]. Artemether, a peroxide sesquiterpenoid lipid-soluble derivative of artemisinin has also been intensively used as an antimalarial and antifever drug [12–14] and in preclinical models was reported to cross the blood-brain barrier [18]. Artemether is also characterized by potent anticancer [15, 16], antiallergic, anti-inflammatory [17, 18], antiviral [19], and antiparasitic pharmacological activities [20, 21]. Cumulative evidences indicate that artemisinin and its derivatives can decrease oxidative stress [19–22]. For example, we reported that artemisinin conferred neuroprotection to PC12 and cortical neuronal cultures from aponecrotic cell death induced by either hydrogen peroxide (H_2_O_2_) or sodium nitroprusside (SNP) oxidative stress insults [21, 22]. This neuroprotective effect was expressed by a significant reduction of the intracellular ROS levels, reduction of caspase 3 activity, and correction of the mitochondrial membrane potential.

The purpose of present study was to investigate the neuroprotective effects of Artemether in AD models in vitro and in vivo, using neuronal PC12 cell cultures exposed to A*β*_1-42_-induced cell death, and in the 3xTg-AD mouse model. We found that Artemether treatment reduced the production of ROS, corrected mitochondrial membrane potential, and conferred neuroprotection by inhibiting aponecrosis of the neurons. A temporal correlation was found between Artemether conferred neuroprotection towards A*β*-induced neurotoxicity and stimulation of AMPK/GSK3*β* phosphorylation activity and increased expression of Nrf2. In 3xTg-AD mice, Artemether attenuated learning and memory deficits, inhibited cortical cell neuronal apoptosis and glial activation, inhibited oxidative stress, and reduced A*β* deposition and phosphorylation of tau protein. Moreover, Artemether induced phosphorylation of the AMPK/GSK3*β*(ser9) pathway which activated the Nrf2 signaling pathway. These findings propose that Artemether could be a new AMPK/GSK3*β*(ser9)/Nrf2 activator for the treatment of inflammation and oxidative stress and a potential therapeutic agent for Alzheimer's disease.

## 2. Materials and Methods

### 2.1. Materials

Fetal bovine serum (FBS), bovine serum albumin (BSA), Dulbecco's modified Eagle's medium (DMEM), and 0.25% trypsin were obtained from GIBCO™ (Grand Island, NY, USA). Artemether, penicillin/streptomycin, Lipofectamine^R^ 2000 reagent, DMSO, and A*β*_1-42_ were obtained from Sigma-Aldrich (St. Louis, MO, USA). Sodium azide (NaN_3_) was obtained from Acros Organic, (New Jersey, USA), and 3-(4,5-dimethylthiazol-2-yl)-2,5-diphenyl tetrazolium bromide (MTT), CellROXs Deep Red Reagent, Hoechst 33342, and 5, 5′,6,6′-tetrachloro-1, 1′,3,3′-tetraethyl-benzimidazolyl-carbocyanineiodide (JC-1) were obtained from Molecular Probes (Eugene, OR, USA). Pierce BCA Protein Assay Kit and Halt™ Protease and Phosphatase Inhibitor Cocktail were purchased from Thermo Scientific (Rockford, IL, USA), The Nissl staining solution, TUNEL kit, Caspase 3 assay kit, a total superoxide dismutase (SOD) assay kit, and lipid peroxidation malonaldehyde (MDA) assay kit were obtained from Beyotime Institute of Biotechnology, Shanghai, China. Anti-phospho-AMPK, anti-AMPK, anti-phospho-GSK3 *β*(ser9), anti-GSK3 *β*, anti-Nrf2, anti-HO-1, anti-phospho-tau, anti-tau, cleaved caspase 3, *β*-amyloid, anti-GFAP, and anti-*β*-actin antibodies were purchased from Cell Signaling Technology (Woburn, MA, USA). Anti-Bax, anti-Bcl2, Iba-1, IL-18, and NF-*κ*B antibodies were purchased from Signalway Antibody (College Park, Maryland, USA). Anti-Rabbit IgG HRP-conjugated secondary antibody was purchased from Promega (Madison, WI, USA), and anti-mouse Alexa 488 was obtained from Invitrogen Co. (Guangzhou, China).

### 2.2. Cell Cultures

PC12 cells were obtained from the National Institute of Child Health and Human Development, NIH, Bethesda, USA. The cells were grown in DMEM supplemented with 10% fetal bovine serum (FBS), 100 *μ*g/ml streptomycin, and 100 units/ml penicillin and maintained at 37° C in a humidified atmosphere of 5% CO_2_. The medium was changed every 3-4 days, and the cell cultures were propagated to new flasks once a week. SH-SY5Y cells, from American Type Culture Collection (ATCC), USA, were cultured in the same way as PC12 cells. Primary cultured neurons were prepared and grown according to the commonly used protocols in our laboratory [23]. Briefly, primary cultured neurons were isolated from the fetal cerebral cortex of the pregnant C57BL/6J mice (Animal facility of Faculty of Health Sciences, University of Macau). The animal care, use, and treatment in this study were strictly in accordance with the University guidelines for the care and use of laboratory animals as defined by the NIH Guide for the Care and Use of Laboratory Animals and Experiments.

### 2.3. Animals Experiments

The homozygous 3xTg-AD mouse (34,830-JAX) was purchased from The Jackson Laboratory. Mice were homozygous for three mutant alleles: homozygous for the coinjected APPSwe and tauP301L transgenes (Tg(APPSwe, tauP301L)1Lfa) and homozygous for the Psen1 mutation. Mice were raised in the Animal Facility of the University of Macau, and all procedures involving animals were approved by the Animal Ethics Committee of the University of Macau (protocol No. UMAEC-039-2015). Adult 3xTg-AD mice (9 months old) were housed in a dedicated germ-free room at 24-26° C, 12 hours/dark light cycle with water and food supplied ad libitum. Mice were randomized into four groups of 10 mice each: group 1: wild-type group; group 2: 3xTg control group; group 3: 3xTg-AD mice treated with a low dose of 5 mg/kg Artemether; and group 4: 3xTg-AD mice treated with a high dose of 20 mg/kg Artemether. Artemether (purity ≥ 98%) was administered by intraperitoneal injection once a day, for four weeks.

### 2.4. Measurement of Cell Viability Using MTT Assay

The cell viability of PC12 cells was measured by MTT assay according to the commonly used protocols in our laboratory [24]. Briefly, PC12 cells were plated on 96-wells (5 × 10^3^ cells/well). Serum-starved cells were treated for 2 hours with Artemether at different concentrations. Thereafter, the medium was removed, and the cells were exposed to 1 *μ*M A*β*_1-42_ for 24 hours in regular medium. MTT was added, and the cells were further cultured for 3 hours at 37° C until crystals of formazan were formed. The medium containing MTT was then removed, and 100 *μ*l of DMSO solution was added to dissolve the crystals. The absorbance of each well was recorded at 570 nm using a BIO-RAD 680 microplate reader (Thermo Fisher, USA). Cell viability was calculated as a percentage of the drug-treated group compared to the control group.

### 2.5. Measurement of Necrotic Cell Death Using LDH Assay

The cytotoxicity of the cells was evaluated by measuring the activity of lactate dehydrogenase (LDH), according to the commonly used protocols in our laboratory [24]. Briefly, PC12 cells were plated on 96-wells (1 × 10^4^ cells/well). After treatment with the drug, the activity of LDH released in the medium was determined, and the released LDH values (%) were normalized to the control group.

### 2.6. Measurement of Reactive Oxygen Species (ROS)

Intracellular reactive oxygen species (ROS) levels were assessed by CellROXs Deep Red Reagent (Thermo Fisher Scientific, USA), according to the commonly used protocols in our laboratory [24]. Briefly, cells grown in 96-well plates were incubated with 1 *μ*M A*β*_1-42_ with or without pretreatment with different concentrations of Artemether. Cells were then incubated with CellROXs Deep Red Reagent (5 *μ*M in fresh DMEM for 1 hour in the dark). Fluorescence was measured, and semiquantitative ROS levels and normalized ROS levels are presented as a percentage of the control group.

### 2.7. Measurement of Mitochondrial Membrane Potential (*△ψ*m)

JC-1 staining dye was used to measure changes in the mitochondrial membrane potential and cellular metabolic activity, according to the commonly used protocols in our laboratory [24]. Briefly, PC12 cells were applied into 96-well plates (1 × 10^4^ cells/well). After drug treatment, cells were incubated with 10 *μ*g/ml JC-1 dye in fresh medium for 30 minutes at 37°C in the dark, washed, and the intensity of red fluorescence and green fluorescence was estimated. The mitochondrial membrane potential (*Δψ*m) was measured as the ratio of the red/green fluorescence intensity and normalized compared to the control group.

### 2.8. Measurement of Cell Apoptosis Using Hoechst 33342 and TUNEL Staining

PC12 cells plated on 96-wells, after drug treatment, washed with 1× PBS and then fixed on ice with 4% paraformaldehyde for 10-15 minutes. The fixed cells were then washed and stained with Hoechst 33342 (10 *μ*g/ml) in PBS for 5 minutes at room temperature. After gently washing twice, cell images were acquired, and the cell apoptosis counts were measured, and the percentages in experimental groups were calculated compared to control groups [24]. TUNEL staining was performed using a TUNEL kit.

### 2.9. Measurement of Apoptosis Using Caspase 3 Activity Assay

The activity of caspase 3 was measured using a commercial Caspase 3 kit (C1116, Beyotime) according to the manufacturer's protocol. Briefly, after drug treatment, PC12 cell cultures were extracted with lysis buffer and centrifuged at 12,500 g, 4°C for 10-15 minutes. Thereafter, detection solution (sample : lysis buffer : Ac − DEVD − pNA = 5 : 4 : 1) was added and incubated for 2 h at 37°C. The absorbance of each well was recorded at 405 nm using a BIO-RAD 680 microplate reader (Thermo Fisher). All values of caspase 3 activity (%) were normalized compared to the control group [24].

### 2.10. Measurement of Apoptosis Using Fluorescence-Activated Cell Sorter (FACS)

PC12 cells (5 × 10^5^ per well) were plated into 6-well dishes. After drug treatment, cell cultures were collected and washed two times with the ice-cold PBS and suspended in the binding buffer. Then, the cell pellet was stained with Annexin-V-FITC solution (5 *μ*l) and propidium iodide (PI) solution (10 *μ*l). The apoptotic cells were analyzed using a FACS instrument (BD AccuriC6, BD, USA) [25].

### 2.11. Measurement of Protein Level and Phosphorylation by Western Blotting

Brain tissues, from mice treated or untreated with drugs, were harvested, washed with ice cold PBS, and lysed in immunoprecipitation assay buffer (RIPA) containing freshly added proteases and phosphatases inhibitors from a cocktail purchased from Thermo Scientific, USA. The neuronal cultures were lysed with 1× sample lysis buffer. The lysate was centrifuged at 13,000 rpm for 15 min. The protein quantification was performed with the BCA protein assay kit, according to the manufacturer's instructions. Proteins were resolved by SDS-PAGE and transferred to a PVDF membrane. Membranes were blocked in 5% nonfat milk in 1× TBST (1 l: Tris-base 2.42 g, NaCl 8.01 g, Tween-20 1 ml, pH = 7.6) for 1 hour. Primary antibodies were used at a 1 : 1000 dilution using 1% BSA in 1× TBST, and the membranes were incubated at 4°C for overnight. Membranes were thereafter washed three times with 1× TBST and then incubated with a secondary antibody at a dilution of 1 : 5000, for 1 hour at room temperature, and immunoblotting was performed using ECL detection kit reagent [26].

### 2.12. Measurement of Mouse Memory Using the Morris Water Maze Test

The Morris water maze consisted of a circular pool of 120 cm in diameter and 40 cm in height and a movable platform with a diameter of 8 cm and four equidistant marking points on the wall of the pool, which served as the water inlet point for the mice and four subbasins. The water temperature was at 22~26°C during the experiment. One hour before the experiment, the mice were moved to the behavior room, allowing them to adjust to the new surroundings. Four days before each memory experiment, the mice were submitted for a place navigation test, and on the fifth day for a spatial probe test [27]. When positioning the navigation experiment, the platform was placed in the middle of one of the quadrants, about 1 cm from the water surface. An experiment was conducted every morning, the latency of finding the platform was recorded by the camera system and the software acquisition system. During the acquisition phase, the mean latency and distance moved were counted as indicators for judging the learning ability of the mice. Twenty-four hours after the end of the navigation test, the mouse was allowed to search the platform for 60 s (the platform was removed). The percent time and cross times spent by mice in target quadrant were recorded. All data acquisition and processing were done by the Morris water maze image automatic monitoring and processing system (ZS Dichuang, Beijing, China).

### 2.13. Preparation of Brain Slices

At the end of the experiment, the mice were anesthetized with pentobarbital sodium (50 mg/kg) followed by transcardial perfusion with 1× PBS. The right hemisphere was frozen at -80°C immediately after dissection, and the left hemisphere was fixed in 4% paraformaldehyde for 24 h, followed by paraffin embedding. Four-micrometer- (4 *μ*m-) thick tissue sections were sliced and kept at 4° C for histological analysis.

### 2.14. Measurements of Apoptosis in Brain Slices Using Nissl Staining and TUNEL Assay

Nissl staining was used to detect surviving neurons. The sections were stained with Nissl staining solution according to the manufacturer's instructions. The survival index was calculated as follows: Survival index (%) = (number of surviving neurons/total number of neurons) × 100%. The TUNEL assay was performed using a TUNEL kit. The processed samples were incubated with a TUNEL reaction mixture for 1 h at 37°C in the dark. The TUNEL-positive cells were observed under a microscope. The apoptosis index in the cortex area was calculated as follows: Apoptosis index (%) = (apoptotic neurons/total neurons) × 100% [28].

### 2.15. Determination of Lipid Peroxidation and Superoxide Dismutase (SOD) Activity in Brain Samples

The activity of SOD and MDA was measured using commercial kits according to the manufacturer's protocol. The brain tissues were extracted with RIPA lysis buffer. A total superoxide dismutase (SOD) assay kit (S0103, Beyotime) and lipid peroxidation malonaldehyde (MDA) assay kit (S0131, Beyotime) were used to measure SOD activity and MDA contents, respectively [28].

### 2.16. Immunohistochemistry (IHC) of Brain Slices to Visualize A*β* and Tau Proteins

Before staining, four-micrometer- (4 *μ*m-) thick tissue sections were subsequently dewaxed, and endogenous peroxidase was quenched with 3% H_2_O_2_. Thereafter, nonspecific binding was blocked by incubating the slides with 10% bovine serum albumin at 37°C for 60 min. Then, tissue sections were incubated with corresponding antibody (A*β*, 1 : 100; phospho-tau, 1 : 200) in PBS containing 1% BSA, at room temperature for 1 h before being placed at 4°C overnight. The following day, the tissue sections were treated with second antibody and followed by DAB color development. DAB-positive cells colored brown; negative control tissue was represented by tissue treated with PBS. The quantitative analysis was conducted by Image-Pro Plus 5.0 [29].

### 2.17. Visualization of Glia Cells by Immunofluorescence (If) Using GFAP Antibody and Immunochemistry (IHC) Using Iba-1 Antibody

4 *μ*m thick tissue sections were subsequently dewaxed. Before staining, nonspecific binding was blocked by incubating with 10% BSA at 37°C for 60 min. Tissue sections were after that incubated overnight with the corresponding antibody (GFAP, 1 : 200, Iba-1, 1 : 100) in PBS containing 1% BSA at 4° C overnight. The following day, the tissue sections were then treated with anti-mouse Alexa 488 antibody for 60 min at room temperature. All incubations were followed by three washes in PBS for 15 min. Nuclei were counterstained with DAPI, and the images were acquired with a Nikon A1 confocal microscope. The quantitative analysis was conducted by Image-Pro Plus 5.0. For the immunochemistry (IHC), the following day, the tissue sections were treated with second antibody and followed by DAB color development. DAB-positive cells colored brown; negative control tissue was represented by tissue treated with PBS.

### 2.18. Statistical Analysis

All the data were presented as mean ± SEM. Each experiment was performed in triplicate. Statistical differences were analyzed by one-way ANOVA in combination with Tukey's post hoc test, and *p* values < 0.05 were regarded as statistically significant. (For the MWM test, escape latency times and distance swam in the hidden platform trial were analyzed via two-way ANOVA with repeated measures.)

## 3. Results

### 3.1. Artemether Attenuated Neurotoxicity Induced by A*β*_1-42_ in Different Neuronal Cell Cultures

**Figure 1:**
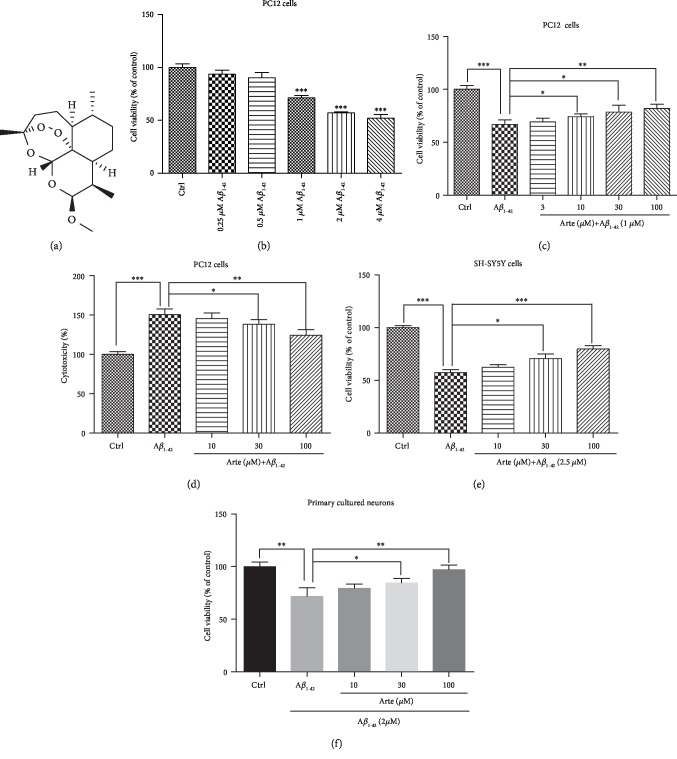
Artemether attenuated A*β*_1-42_-induced neurotoxicity in neuronal cell cultures. (a) Chemical structure of Artemether. (b–d) Neurotoxicity of A*β*_1-42_ in PC12 cells and Artemether-induced neuroprotection. PC12 cells were grown in 96-well plates and treated for 24 h with different concentrations of A*β*_1-42_; cell viability was analyzed by (b) MTT assay. ^∗∗∗^*p* < 0.001 versus control. Cells were pretreated with Artemether at an indicated concentration or 0.1% DMSO (vehicle control) for 2 h and then incubated with or without 1 *μ*M A*β*_1-42_ for an additional 24 h. Cell viability was measured by (c) MTT assay and (d) LDH release assay. Results are presented as mean ± SEM (*n* = 3). ^∗^*p* < 0.05, ^∗∗^*p* < 0.01, and ^∗∗∗^*p* < 0.001 were considered significantly different. (e) SH-SY5Y neuroblastoma cells were pretreated with different doses of Artemether for 2 h followed by exposure to 2.5 *μ*M A*β*_1-42_ for an additional 24 h; cell viability was measured by MTT assay. Results are presented as mean ± SEM (*n* = 3). ^∗^*p* < 0.05 and ^∗∗∗^*p* < 0.001 were considered significantly different. (f) Primary brain cortex neuronal cultures were pretreated with different doses of Artemether for 2 h and exposed to 2 *μ*M A*β*1-42 for 24 h; cell viability was measured by MTT assay. Results are presented as mean ± SEM (*n* = 3). ^∗^*p* < 0.05 and ^∗∗^*p* < 0.01 were considered significantly different.

In the first step, we investigated the neurotoxic effect of A*β*_1-42_ in PC12 cell cultures. The cells were incubated with increasing concentrations of A*β*_1-42_ in 96-well plates for 24 h. Thereafter, the cell viability was measured by MTT assay (Figure 1(b)). A*β*_1-42_ induced cell death in a concentration-dependent manner and reached a maximal neurotoxic effect at 4 *μ*M, as previously reported [30]. A dose of 1 *μ*M A*β*_1-42_ which causes about 50% cell death was chosen for further experiments. In the next step, we analyzed whether Artemether (Figure 1(a)) can confer neuroprotection towards A*β*_1-42_-induced cell death. The data presented in Figure 1(c) clearly indicates that Artemether induced neuroprotection in a dose-dependent manner towards A*β*_1-42_-induced cell death. In another approach, by measuring LDH release, we sought to investigate whether Artemether can confer neuroprotection towards A*β*_1-42_-induced necrotic cell death (Figure 1(d)). Similar to the findings in Figure 1(c), Artemether, in a concentration-dependent manner, conferred neuroprotection with an effective dose of 50% (EC_50_) around 10 *μ*M. To extend the possible neuroprotective effect of Artemether to other neurons, we also investigated its effects on SH-SY5Y human neuroblastoma and mice brain cortex primary neuronal cell cultures. In these neuronal cell cultures, Artemether conferred neuroprotection towards A*β*_1-42_-induced cell death in a concentration-dependent manner (although with a higher ED_50_), consistent with its potent neuroprotective effect in PC12 cell model (Figures 1(e) and 1(f)).

### 3.2. Artemether Decreased Apoptosis Induced by A*β*_1-42_ in PC12 Cells

**Figure 2:**
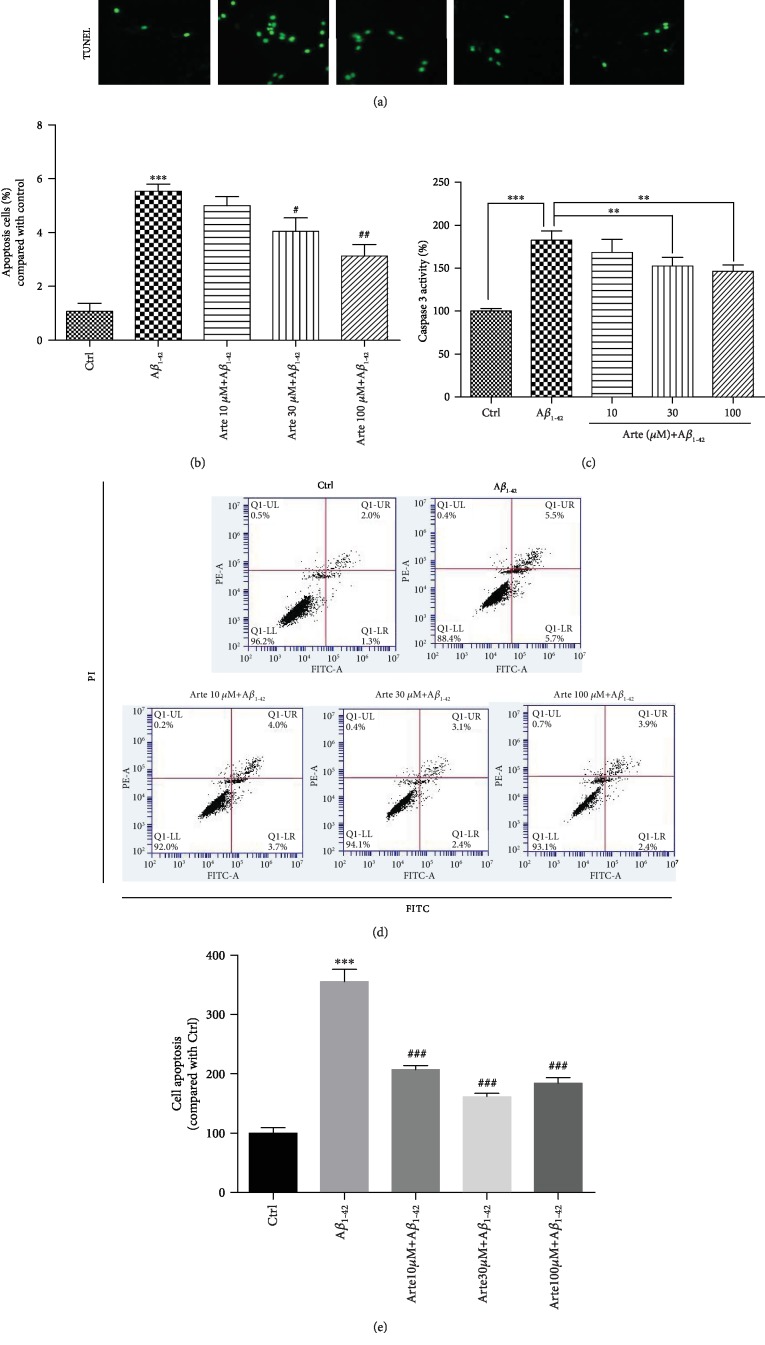
Artemether-induced neuroprotective effect on A*β*_1-42_-induced apoptosis in PC12 cell cultures. (a) PC12 cells were pretreated with different concentrations of Artemether for 2 h followed by exposure of 24 h to 1 *μ*M A*β*_1-42_. Apoptotic cells were observed by Hoechst 33342 staining and TUNEL staining as shown in fluorescence images taken with a fluorescence microscope. The apoptotic cells with condensed chromatin are indicated by an arrowhead. (b) Quantitation of apoptotic cell's nuclei. ^∗∗∗^*p* < 0.001 versus the control group and ^#^*p* < 0.05 and ^##^*p* < 0.01 versus the A*β*_1-42_-treated group were considered significantly different. (c) Experiment performed as in (a), and caspase 3 activity was measured. ^∗∗^*p* < 0.01 and ^∗∗∗^*p* < 0.001 were considered significantly different. (d, e) Experiment performed as in (a), and apoptosis was determined by flow cytometry. All results are presented as mean ± SEM (*n* = 3). ^∗∗∗^*p* < 0.001 versus the control group and ^###^*p* < 0.001 versus the A*β*_1-42_-treated group were considered significantly different.

In order to investigate the neuroprotective effect of Artemether on A*β*_1-42_ -induced apoptosis, Hoechst staining was used for staining the nuclei of cells to distinguish the compact chromatin of apoptotic nuclei. After exposure to A*β*_1-42_ for 24 h, PC12 cell's nuclei become more condensed, and cell morphology changed as shown in Figures 2(a) and 2(b) (arrowheads). Pretreatment for 2 h with different concentrations of Artemether resulted with a significant reduction in the nuclei' condensation. The cell apoptosis were further detected by TUNEL staining (Figure 2(a)). In accordance with these, the increase in caspase 3 upon A*β*1-42 stimulation was decreased by pretreatment with Artemether (Figure 2(c)). And we got similar results in primary neuronal cell cultures (Fig. s1-Hoechst 33342 and TUNEL). Moreover, FACS analyses of Annexin-V-FITC-labeled cells indicated that Artemether inhibited by 60% the apoptosis induced by A*β*_1-42_, (Figures 2(d) and 2(e)), findings consistent with the results of nucleus condensation and caspase 3 activity.

### 3.3. Artemether Reduced Intracellular ROS, Reduced the Decline in Mitochondrial Membrane Potential, and Increased Nrf-2 Protein Expression in A*β*_1-42_-Treated PC12 Cells

**Figure 3:**
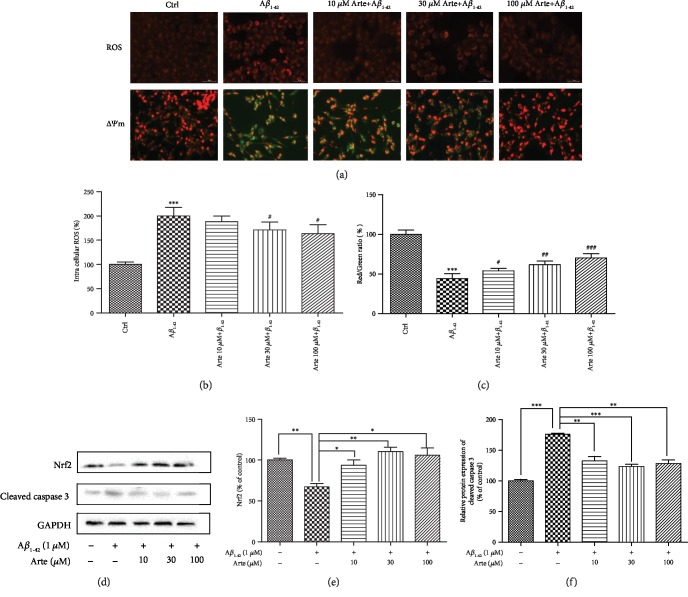
Artemether reduced intracellular ROS, reduced the decline in mitochondrial membrane potential, and increased Nrf-2 protein expression in A*β*_1-42_-treated PC12 cells. (a) PC12 cells pretreated for 2 h with different concentrations of Artemether followed by exposure for 24 h with 1 *μ*M A*β*_1-42_. The fluorescent images represent the intracellular ROS level as determined by the CellROXs Deep Red Reagent and by staining the cells with JC-1 dyes. Fluorescence intensity of images depicts the change of mitochondrial membrane potential (*△ψ*m). (b) Quantitation of the percentage of the intracellular ROS level. Results are presented as mean ± SEM (*n* = 3). ^#^*p* < 0.05 versus the A*β*_1-42_-treated group and ^∗∗∗^*p* < 0.001 versus the control group were considered significantly different. (c) Red to green fluorescence intensity ratio (increase of mitochondrial membrane potential). Three independent experiments were included. ^#^*p* < 0.05 and ^###^*p* < 0.001 versus the A*β*_1-42_-treated group and ^∗∗∗^*p* < 0.001 versus the control group were considered significantly different. (d) PC12 cells pretreated for 2 h with different concentrations of Artemether were incubated with 1 *μ*M A*β*_1-42_, followed by measurements of (d, e) cleaved caspase 3 and (f) Nrf2 expression levels, determined by western blotting followed by quantitation of protein bands relative to GAPDH. Three independent experiments were included. ^∗^*p* < 0.05, ^∗∗^*p* < 0.01, and ^∗∗∗^*p* < 0.001 were considered significantly different.

Treatment with 1 *μ*M A*β*_1-42_ for 24 h increased by 2.3-fold the production of intracellular ROS compared with that of control group, and this increase was attenuated 1.6-fold by the pretreatment with 10-100 *μ*M Artemether (Figures 3(a) and 3(b)). These results indicate that Artemether's neuroprotective effect is mediated in part by its antioxidant activities. And we got similar results in primary neuronal cell cultures (Fig. [Supplementary-material supplementary-material-1]).

Mitochondrial function, a pivotal indicator of cell health, can be evaluated by monitoring changes in mitochondrial membrane potential (*△ψ*m) using JC-1 dye. In healthy cells, the dye accumulates in the mitochondria as red fluorescent aggregates. In apoptotic cells, the dye is converted to green fluorescent monomers and remains in the cytoplasm. Therefore, the red/green fluorescence ratio can be used in determining the mitochondrial function of A*β*_1-42_‑induced PC12 cells with or without pretreatment with Artemether. After exposure of PC12 cells to 1 *μ*M A*β*_1-42_ for 24 h, *△ψ*m was significantly decreased by 60% (Figures 3(a), JC-1, and 3(c)). The decrease in red fluorescence intensity and an increase in green fluorescence intensity imply the decrease of *△ψ*m. The decreased *△ψ*m by A*β*_1-42_ was corrected (increased) by pretreatment with Artemether, as measured by red/green fluorescence intensity ratio (Figure 3(c)). These results indicated that Artemether treatment corrected mitochondrial dysfunctions induced by A*β*1-42. And we got similar results in primary neuronal cell cultures (Fig. [Supplementary-material supplementary-material-1]).

The transcription factor Nrf2 allows for adaptation and survival under stress by regulating gene expression in different networks of cytoprotective proteins, including anti-inflammatory and antioxidant, and proteins that help repair or remove damaged macromolecules. Nrf2 plays a crucial role in maintaining cellular redox homeostasis and controlling mitochondria to produce the reactive oxygen species. Nrf2 affects the *△ψ*m, ATP synthesis, lipid peroxidation, and activation of Nrf2 under stress or growth factor stimulation to counteract the increase of ROS production in mitochondria, contributing to neuroprotection [31, 32]. With this background, we investigated the temporal relationship between the effect of Artemether on Nrf2 protein expression and apoptosis executor, caspase 3 activity in A*β*_1-42_-treated PC12 cells (Figures 3(d)–3(f)). The results indicate that A*β*_1-42_ decreased significantly by 40% and increased by 75% the expression levels of Nrf2 and caspase 3 proteins, while pretreatment for 2 h with 10-100 *μ*M Artemether reversed these effects—increased by 50-60% of Nrf2 protein levels and decreased by 50% of caspase 3 protein levels. This indirect relationship may explain the neuroprotective effects of Artemether towards A*β*_1-42_-induced apoptosis in PC12 cells.

### 3.4. Involvement of the AMPK/GSK3*β*(ser9) Signaling Pathway in the Neuroprotective Effects of Artemether

**Figure 4:**
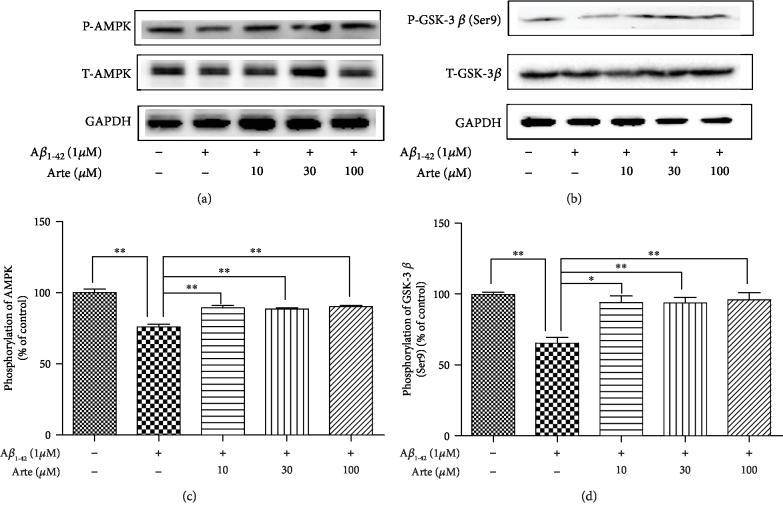
Artemether increased phosphorylation of AMPK/GSK3*β*(ser9)in PC12 cell cultures exposed to A*β*_1-42._ (a, c) PC12 cells pretreated for 2 h with different concentrations of Artemether were incubated with 1 *μ*M A*β*_1-42_ for 24 h .The lysates were submitted for measurements of phosphorylations by western blotting using specific antibodies for AMPK (P-AMPK), total AMPK (T-AMPK), GSK3*β*(ser9) (P-GSK3*β*), and total GSK3*β* (T-GSK3*β*). GAPDH was used for normalization. Three independent experiments were included. (b, d) Quantitation of phosphorylated kinases. ^∗^*p* < 0.05 and ^∗∗^*p* < 0.01 were considered significantly different.

AMP-activated protein kinase (AMPK), a major regulator of cellular energy homeostasis which plays a central role in lipid and glucose metabolism, is also involved in the pathogenesis of Alzheimer's disease (AD) [33]. It was also reported that brain cortical neuroprotection might be mediated by enhancing the Ser 9 phosphorylation of glycogen synthase kinase3*β* (GSK3*β*) [34]. For this reason, we sought to investigate the phosphorylation activities of AMPK/GSK3*β*(ser9) in relation to the neuroprotective effects of Artemether. PC12 cell cultures were treated with 10-100 *μ*M of Artemether; the cultures were lysed and extracts submitted for western blotting using respective anti-pan and anti-phospho antibodies for AMPK (Thr 172) and GSK3*β* (Ser 9). As expected [33, 35], the phosphorylation of AMPK/GSK3*β* was decreased by 25-40% after A*β*_1-42_ treatment (insult), compared with the control group. Artemether pretreatment of A*β*_1-42_-treated PC12 cell cultures changed the phosphorylation of AMPK and GSK3*β* which were significantly increased, closed to phosphorylation values in control untreated cultures (Figure 4).

**Figure 5:**
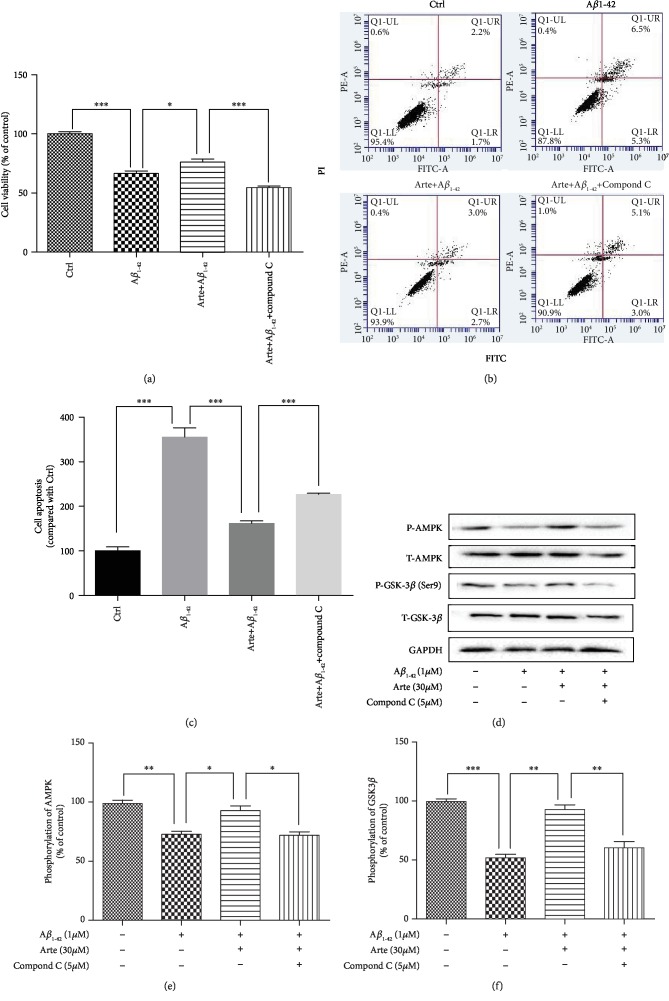
Direct relationship between Artemether effects on AMPK/GSK3*β*(ser9) phosphorylation and neuroprotection towards A*β*_1-42_-induced apoptotic cell death in PC12 cells. PC12 cells pretreated with 5 *μ*M compound C (AMPK inhibitor) for 30 min, followed by incubation with 1 *μ*M A*β*_1-42_ in the presence or absence of different concentrations of Artemether. (a) Cell viability was measured by MTT assay. ^∗^*p* < 0.05 and ^∗∗∗^*p* < 0.001 were considered significantly different. (b) Apoptosis was determined by flow cytometry. (c) Quantitation of the percentage of cell apoptosis. ^∗∗∗^*p* < 0.001 was considered significantly different. (d) Phosphorylation activities of AMPK/GSK3*β*(ser9). GAPDH was used for normalization. (e, f) Quantitation of phosphorylation activities of AMPK/GSK3*β*. All results are presented as mean ± SEM (*n* = 3); ^∗^*p* < 0.05, ^∗∗^*p* < 0.01, and ^∗∗∗^*p* < 0.001 were considered significantly different.

Compound C (also called dorsomorphin), a cell-permeable, potent, relative selective, and reversible ATP-competitive inhibitor of AMPK, has been widely used in cell-based, biochemical, and in vivo assays as an AMPK inhibitor [36]. Therefore, we next questioned if Artemether can confer neuroprotection in PC12 cell cultures treated with compound C to verify the involvement of AMPK in Artemether-induced neuroprotection towards A*β*_1-42_-induced cell death in PC12 cell cultures. Cells were pretreated with the 5 *μ*M compound C for 30 min in the presence or absence of 30 *μ*M Artemether (2 h) and further incubated with 1 *μ*M of A*β*_1-42_ for 24 h. Thereafter, cell viability was measured by MTT assay (Figure 5(a)), cell apoptosis was measured by Annexin-V-FITC FACS assay (Figures 5(b) and 5(c)) in parallel with measurements of AMPK/GSK3*β*(ser9) phosphorylation activities (Figures 5(d) and 5(f)). The results clearly indicate that compound C treatment significantly inhibited the neuroprotective effects of Artemether, in apparent direct correlation to the decreased AMPK/GSK3*β*(ser9) phosphorylation activities.

### 3.5. Artemether Therapy Attenuated Spatial Learning and Reference Memory Deficits in 3xTg Mouse Model

Based on these in vitro findings, we next verified whether the neuroprotective effects of Artemether found in neuronal cultures can be measured in the triple transgenic mouse model of Alzheimer's disease (3xTg-AD), which exhibits the increase of amyloid-*β* oligomer accumulation in an age-dependent manner and extracellular plaques and tau pathology in the brain paralleled by learning and memory impairment [37].

**Figure 6:**
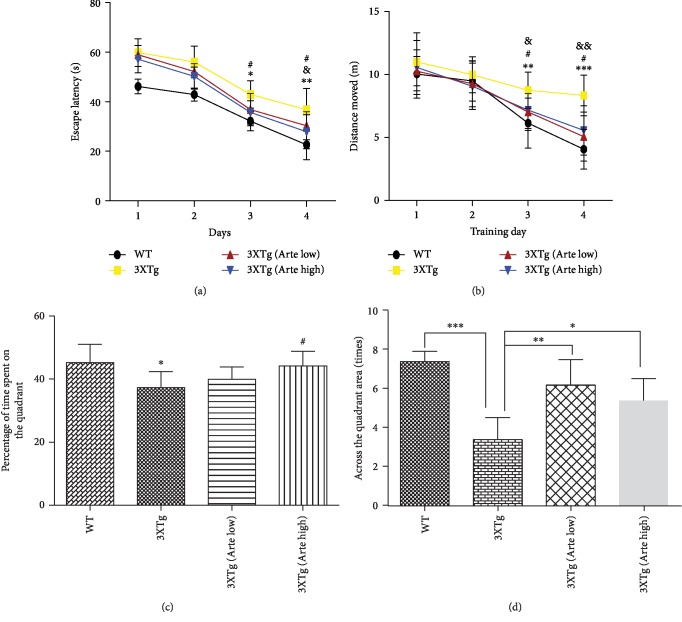
The effect of Artemether treatment on spatial learning and reference memory in 3xTg-AD mice. Artemether was administered by intraperitoneal injection once a day, at low doses of 5 mg/kg and high doses of 20 mg/kg for 4 weeks. (a, b) Effect on time needed to find the hidden platform (escape latency) and distance moved during the hidden platform trial. ^∗^*p* < 0.05, ^∗∗^*p* < 0.01, and ^∗∗∗^*p* < 0.001, 3xTg group versus WT group; ^#^*p* < 0.05, 3xTg (Arte high) versus 3xTg group; and ^&^*p* < 0.05 and ^&&^*p* < 0.01, 3xTg (Arte low) versus 3xTg group, were considered significantly different; ten mice per group were used for the Morris water maze. (c, d) Effect on the percent time and cross times spent by mice in a target quadrant in the probe trial. ^∗^*p* < 0.05 versus the WT group and ^#^*p* < 0.05 versus the 3xTg group were considered significantly different; ten mice per group were used for the Morris water maze.

In the first phase, to evaluate whether Artemether could ameliorate cognitive function impairments in 3XTg-AD mice, a Morris water maze test was conducted (Figure 6). In the Morris water maze training, mice were requested to find hidden platforms under the water. Performance was evaluated by the average time required to find the escape platform (escape latency) and distances swam in each group. Then, a probe trial was performed on the fifth day. Performance was evaluated by comparing the percent time spent in the target quadrant and cross times spent in the target quadrant by mice. As previously demonstrated [38], untreated 3xTg-AD mice showed significant impairment in learning this task compared with wild-type mice. Indeed, untreated 3xTg-AD mice took longer to find the hidden platform, and distance swam was longer, thus confirming the memory impairment. Artemether treatment, at both low and high doses, significantly decreased the escape latency, shortened swimming distance of 3xTg-AD mice compared to the untreated 3xTg-AD mice group (Figures 6(a) and 6(b)). Treatment with the Artemether significantly increased the cross times and the percentage of time spent on the target quadrant (Figures 6(b) and 6(c)). These findings indicate that Artemether treatment rescued spatial learning and reference memory deficits in the 3xTg-AD mouse model.

### 3.6. Artemether Inhibited Brain Cortex Apoptosis in 3xTg-AD Mice

**Figure 7:**
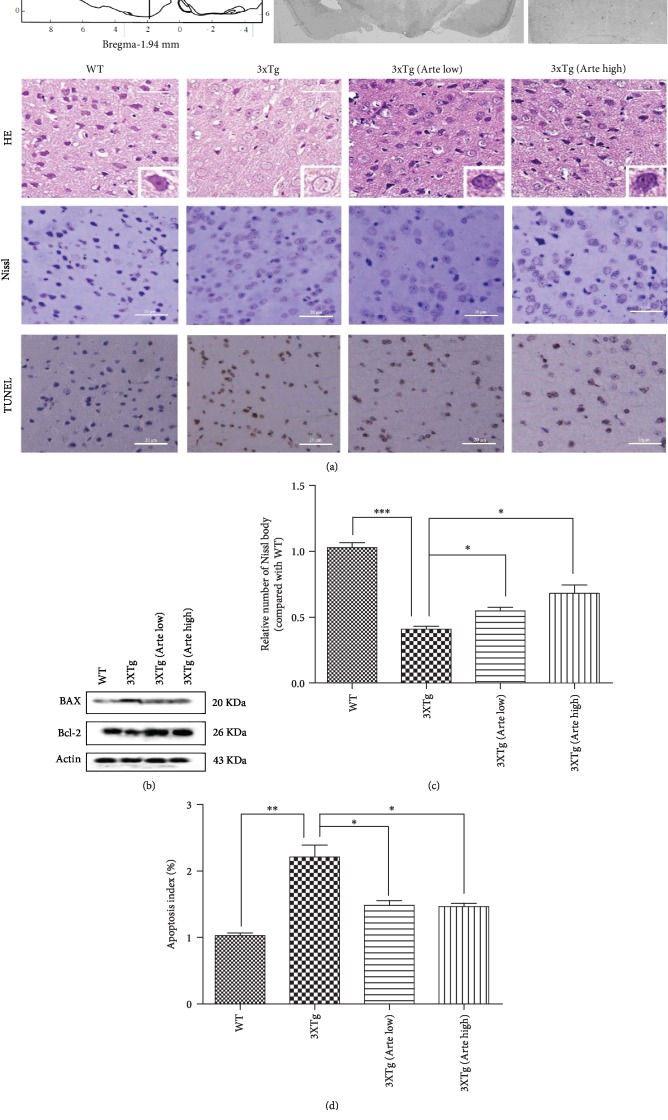
Artemether treatment decreased brain cortex apoptosis in 3xTg-AD mice. Artemether was administered by intraperitoneal injection, once a day, at low doses of 5 mg/kg and high doses of 20 mg/kg for 4 weeks. (a) The HE (gross morphology), Nissl (neuronal viability), and deoxynucleotidyl transferase dUTP nick-end labeling (TUNEL, apoptosis) staining in brain cortex slices of wild-type mice, compared to 3xTg-AD mice without or treated with low (Arte low) or high (Arte high) dose of Artemether (scale bar = 20 *μ*m). Brain cortex area analyzed for Nissl and TUNEL staining is shown in the figure (bregma (1.94 mm), A or B area); five slides per mouse and five mice per treatment group were used for analysis. (b) Western blotting of Bax and Bcl2 protein expression in each group. Three mice per group were used for western blot. (c, d) Quantitation of the Nissl and TUNEL staining data. ^∗^*p* < 0.05, ^∗∗^*p* < 0.01, and ^∗∗∗^*p* < 0.001 were considered significantly different.

In the next phase, to investigate the apparent correlation between Artemether improvement of cognition and neuroprotection from amyloid plaques and neurofibrillary tangle-like pathology, predominantly expressed in the cerebral cortex [39], we sought to histochemically quantify using Nissl and TUNEL staining the neuronal living and apoptotic brain cortical cells, in 3xTg-AD mice treated with Artemether, by comparison to untreated 3xTg-AD mice (Figure 7). First, the neurons of the cortex were characterized by Hematoxylin-Eosin (HE) staining. In wild-type mice, they presented a typical organization with round shape and clear nuclei, while in the cortex of 3xTg-AD mice, their morphology indicated shrinkage of nuclei and apoptotic signs. Artemether was found to partially correct these pathological features, as demonstrated by improved shape and organization of neurons (Figure 7(a), HE). Consistent with HE observation, severe deformation and disorganization of neurons were observed in the 3xTg-AD mouse brain cortex, with the significant reduction of the relative number of Nissl-positive neurons (Figures 7(a), Nissl, and 7(c)). Artemether treatment of 3xTg-AD mice increased the number of Nissl bodies of cortical neurons in a concentration-dependent manner (Figures 7(a), Nissl, and 7(c)). Reducing the apoptosis of brain neurons is an important requirement for neuroprotective therapy of Alzheimer's disease. To observe the effect of Artemether on brain cortical neuronal apoptosis, TUNEL staining (Figures 7(a), TUNEL, and 7(d)) and western blotting for Bax (apoptosis promoter) and Bcl-2 (apoptosis inhibitor) (Figure 7(b)) were performed. As depicted in Figures 7(a), TUNEL, and 7(d), the number of apoptotic brain cortical neurons was significantly decreased following treatment of 3xTg-AD mice with Artemether, relative to untreated 3xTg-AD mice. As depicted in Figure 7(b), it was also noticed that Artemether treatment significantly increased the expression of Bcl-2 and decreased the expression of Bax, when compared to untreated 3xTg-AD mice. These results may indicate that Artemether reduced brain cortical neuronal apoptosis by affecting the balance of apoptotic proteins.

### 3.7. Artemether Attenuated Oxidative Stress in the Brain Cortex of 3xTg-AD Mice

**Figure 8:**
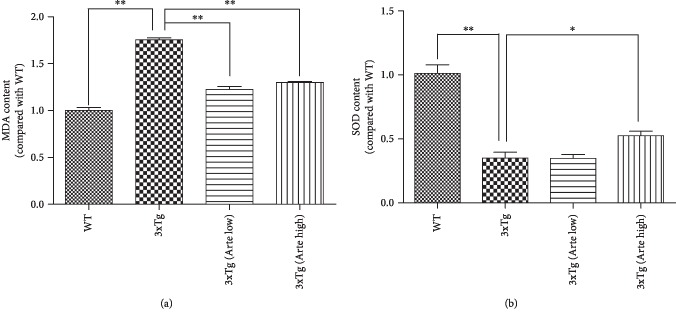
Artemether decreased the level of malonaldehyde (MDA) and increased the level of superoxide dismutase (SOD) in the brain cortex of 3xTg-AD mice. Artemether was administered to mice by intraperitoneal injection, once a day, at low doses of 5 mg/kg and high doses of 20 mg/kg, for 4 weeks. The levels of malonaldehyde (MDA) (a) and superoxide dismutase (SOD) (b) were determined using an enzyme-linked immunosorbent assay in wild-type (WT) compared to 3xTg-AD mice treated with either a low (Arte low) or high (Arte high) dose of Artemether or untreated (3xTg). ^∗^*p* < 0.05 and ^∗∗^*p* < 0.01 were considered significantly different. Five mice per group were used for analysis.

Oxidative stress is an early event in AD pathogenesis, which can precede the formation of A*β* deposits. Therefore, quantitation of the byproducts of oxidative biomolecular damage such as malondialdehyde (MDA), a typical marker of lipid peroxidation and superoxide dismutase (SOD), an antioxidant enzyme which plays the fundamental role in the antioxidant protective capacity of the brain against free radical insult, is widely adopted to evaluate oxidative stress. The results presented in Figure 8 indicated that both MDA levels and SOD activity were significantly affected in 3xTg-AD mice compared to wild-type mice as previously reported [40]. After Artemether treatment at a high dose, the levels of MDA and SOD in the brain extracts of 3xTg-AD mice were significantly reversed by about 25%. These findings further support the proposal of the antioxidant effects of Artemether in relation to inhibition of brain cortical apoptosis and attenuation of cognitive deficits in 3xTg mice model.

### 3.8. Artemether Reduced Amyloid-*β* Deposition and Phosphorylation of Tau in the Brain Cortex of 3xTg-AD Mice

**Figure 9:**
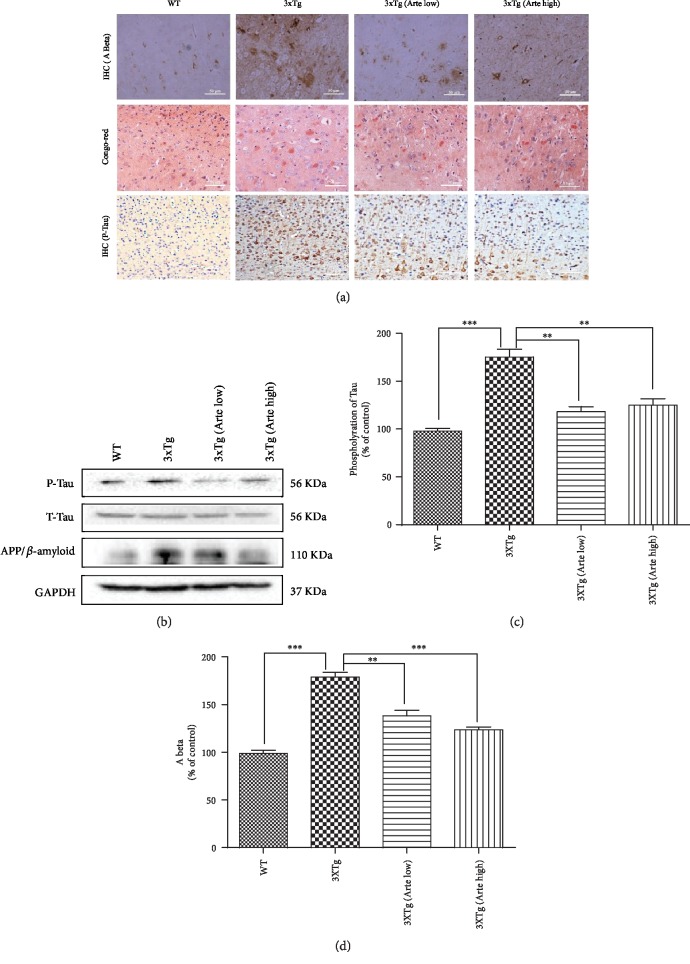
Artemether treatment reduced A*β* deposition and phosphorylation of Tau in the brain cortex of 3xTg-AD mice. Artemether was administered to mice by intraperitoneal injection, once a day, at low doses of 5 mg/kg and high doses of 20 mg/kg, for 4 weeks. Thereafter, the brain cortex of wild-type (WT) compared to that of 3xTg-AD mice treated with either a low (Arte low) or high dose (Arte high) of Artemether or untreated (3xTg). (a) Immunohistochemistry of amyloid-*β*, Congo red staining (label amyloidosis), and phosphorylated tau (scale bar = 50 *μ*m). Brain cortex area analyzed for amyloid-*β*, Congo red staining, and phosphorylated tau was the same as before (Figure 7); five slides per mouse and five mice per treatment group were used for analysis. (b) Western blot of *β*-amyloid and phosphorylation of Tau in each animal group; three mice per treatment group were used for western blot. (c, d) Quantitation of western blots. ^∗∗^*p* < 0.01 and ^∗∗∗^*p* < 0.001 were considered significantly different.

Amyloid-*β* deposition, hyperphosphorylation of tau, and oxidative stress closely interplay in AD [41]. Therefore, we sought to examine immunohistochemically the effect of Artemether therapy on expression of amyloid-*β* (Figures 9(a) and 9(d)) and tau hyperphosphorylation (Figures 9(a)–9(c)) pathology in the brain cortex of 3xTg-AD mice using *β*-amyloid, phospho-tau (ser 416, which has been demonstrated to be a major phosphorylation site of CaMK II in Alzheimer's disease brain) and pan-tau (T-tau) antibodies. As expected, a significant increase of amyloid-*β* deposition and phosphorylation of tau was found in the brain cortex of 3xTg-AD mice. Quantitative analyses of immunoreactivity confirmed the qualitative histological findings (Figures 9(c) and 9(d)). In brains of mice treated with Artemether at both low and high dose, 20-40% reductions in amyloid-*β* deposition and phosphorylation of tau were measured. Similarly, using western blotting with the same antibodies, a significant, 50% reduction of amyloid-*β* and phosphorylated tau protein levels were measured (Figure 9(b)). These biochemical and immunohistochemically analyses indicate a temporal causal relationship between Artemether therapy and reduced amyloid-*β* deposition and phosphorylation of tau pathologies in 3xTg-AD mice.

### 3.9. Artemether Reduced Brain Inflammation in 3xTg-AD Mice

**Figure 10:**
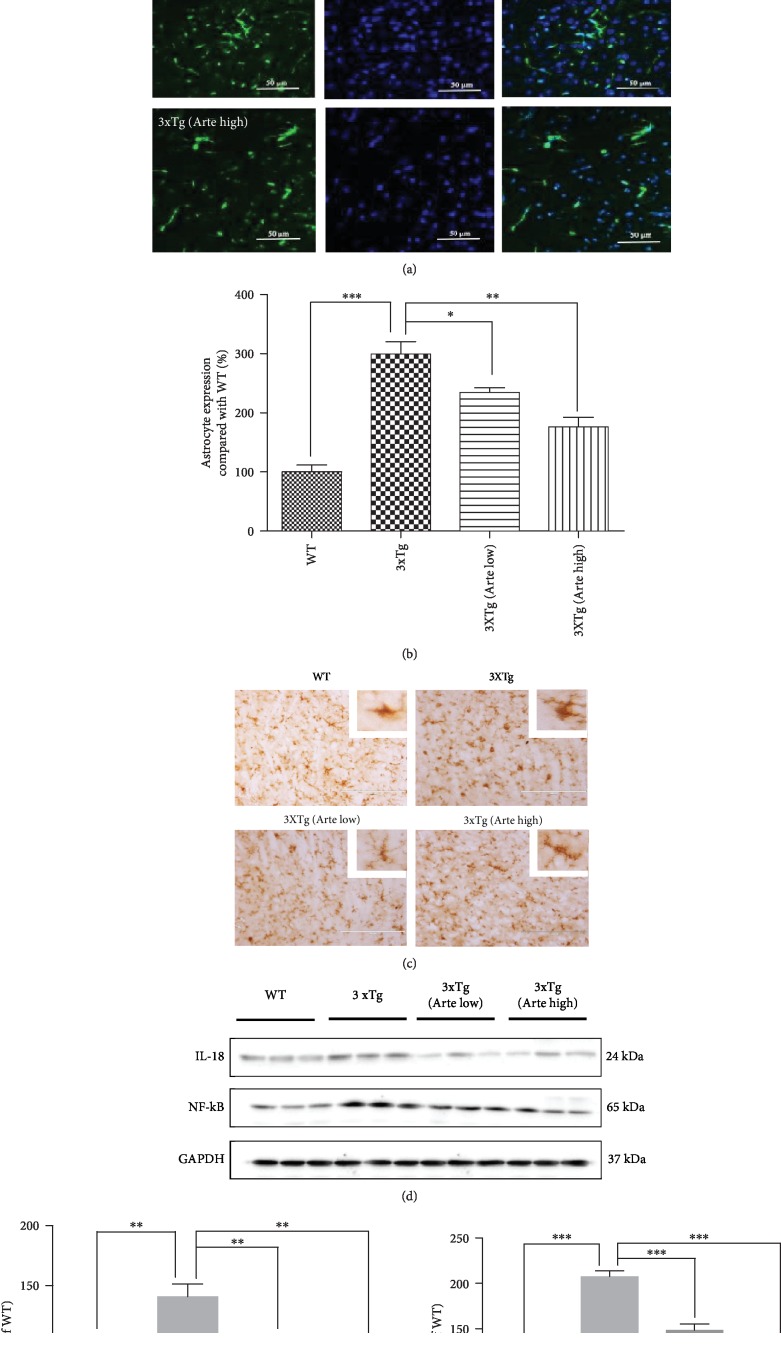
Artemether treatment attenuated gliosis and expression of inflammatory molecules in the brain cortex of 3xTg-AD mice. Artemether was administered to mice by intraperitoneal injection, once a day, at low doses of 5 mg/kg and high doses of 20 mg/kg, for 4 weeks. Thereafter, the brain cortex slices or extracts of wild-type (WT) compared to 3xTg-AD mice treated with either a low (Arte low) or high dose (Arte high) of Artemether or untreated (3xTg) was submitted for analyses. (a, b) GFAP immunohistochemical expression, scale bar = 50 *μ*m. The histograms in (b) represent the percent count of immunoreactive cells compared to wild-type, in the different experimental groups, ^∗^*p* < 0.05, ^∗∗^*p* < 0.01, and ^∗∗∗^*p* < 0.001 were considered significantly different. Brain cortex area analyzed for GFAP and Iba-1 expression level was the same as before; five slides per mouse and five mice per treatment group were used for analysis. (c) Iba-1 immunohistochemical expression, scale bar = 200 *μ*m. (d) Western blot analysis of Artemether effect on IL-18 and NF-*κ*B; three mice per treatment group were used for western blot. (e, f) Quantitation of western blots. ^∗∗^*p* < 0.01 and ^∗∗∗^*p* < 0.001 were considered significantly different.

The involvement of an inflammatory process in the pathophysiology of AD induces microglial activation and release of cytokines including NF-*κ*B activation, essential for maximal cytokine transcription after A*β* stimulation [42]. Glial fibrillary acidic protein (GFAP) is linked with old age and the onset of AD pathology [43]. Therefore, we verified whether the astrocyte of the cerebral cortex was active in 3xTg-AD mice (Figures 10(a) and 10(b)). As expected, the level of GFAP-positive cell population was 3-fold higher in the 3xTg-AD mice, compared with the wild-type mice. Artemether treatment, at both low and high doses, significantly decreased GFAP-positive population, suggestive of an anti-inflammatory effect. Further, we verified whether the microglia of cerebral cortex was active in 3xTg-AD mice, Iba 1 (or marker of microglia) immunostaining was used (Figure 10(c)). As expected, the destruction of microglia morphology in 3xTg mice was improved after Artemether treatment. In support of these findings, Artemether decreased the levels of the brain inflammatory markers IL-18 and NF-*κ*B which were found increased in 3xTg-AD mice (Figure 10(d)), as measured by western blotting analysis of brain extracts. These findings suggest that the therapeutic effect of Artemether in the 3xTg-AD mice is due to both neuroprotective and anti-inflammatory effects.

### 3.10. Artemether Stimulated AMPK/GSK3*β*(ser9)/Nrf2 Signaling in the Brain Cortex of 3xTg-AD Mice

**Figure 11:**
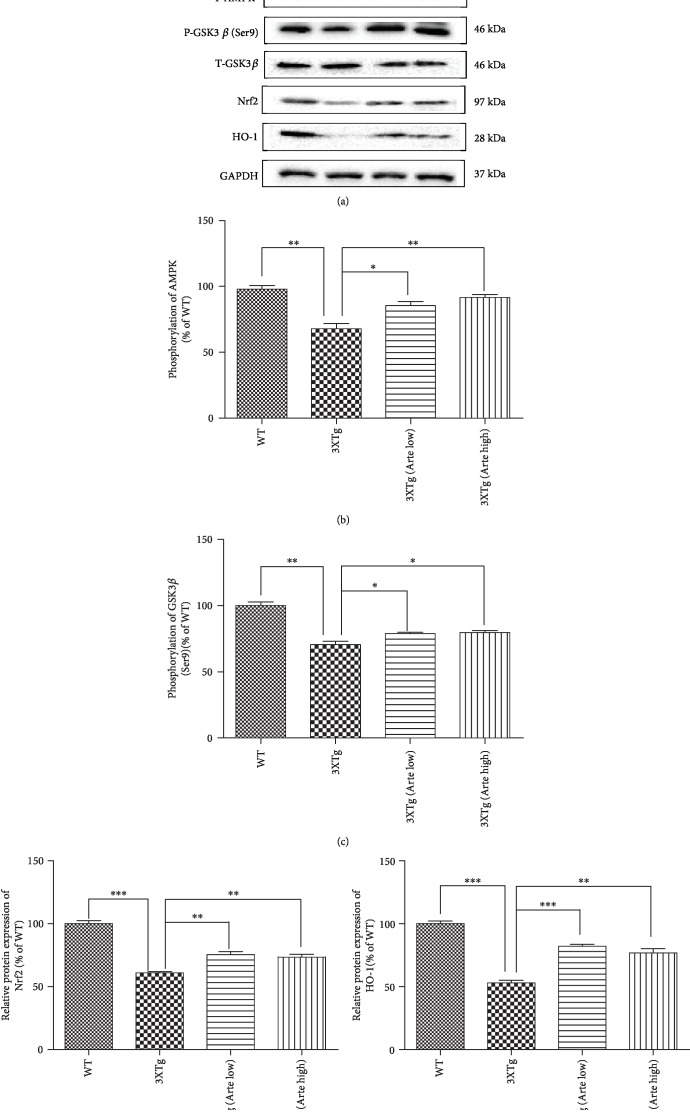
Artemether stimulated the phosphorylation of AMPK/GSK3*β*(ser9) and increased the expression levels of Nrf2 and heme oxygenase-1 (HO-1) in the brain cortex of 3xTg-AD mice. Artemether was administered to mice by intraperitoneal injection, once a day, at low doses of 5 mg/kg and high doses of 20 mg/kg for 4 weeks. Thereafter, the brain cortex extracts of wild-type (WT) compared to 3xTg-AD mice treated with either a low (Arte low) or high dose (Arte high) of Artemether or untreated (3xTg) were submitted for analyses. (a) Phosphorylations of AMPK/GSK3*β*(ser9) and protein expression levels of Nrf2 and heme oxygenase-1 (HO-1); three mice per treatment group were used for western blot. (b–e) Quantitation of western blotting results of the different experimental groups. ^∗^*p* < 0.05, ^∗∗^*p* < 0.01, and ^∗∗∗^*p* < 0.001 were considered significantly different.

It is becoming evident that AMPK and GSK3*β*(ser9) are deregulated in the AD. Since in neuronal cultures treated with Artemether AMPK/GSK3*β*(ser9)/Nrf2 signaling was stimulated in correlation with neuroprotection, we asked whether these activities can be found and measured in brain extracts of Artemether-treated 3xTg-AD mice. Figures 11(a)–11(c) indicate that phosphorylation of AMPK/GSK3*β*(ser9) was decreased by 20-40% in brain extracts of 3xTg-AD mice compared to that of wild-type mice while Artemether therapy increased these phosphorylations by about 50%. Consistent with these findings and results with the neuronal cultures, in brain extracts of Artemether-treated 3xTg-AD mice, an upregulation of the Nrf2/HO-1 antioxidant pathway was measured (Figures 11(a), 11(d), and 11(e)).

## 4. Discussion

Alzheimer's disease (AD) is a neurodegenerative disorder characterized by the accumulation of senile plaques and neurofibrillary tangles in the brain. Considering the multifactorial nature of the AD, the search for Chinese herbal compounds, with a wide spectrum of neuroprotective activities, holds very promising potential for the development of drugs for AD therapy. In our work, for the first time, we have demonstrated the neuroprotection in vivo and in vitro for Artemether, a derivative of artemisinin, for a potential neurotherapeutic application in the AD. Artemether treatment of amyloid-*β*-induced cell death in neuronal cultures reduced the production of ROS, corrected mitochondrial membrane potential, and conferred neuroprotection by inhibiting aponecrosis of neurons. Artemether therapy in 3xTg-AD mice resulted in the recovery of function from cognitive impairment, related to reduced apoptosis, oxidative stress markers, amyloid-*β* deposition, and phosphorylation of tau and improvement of inflammatory markers' parameters in the brain cortex. A temporal correlation was found between Artemether-induced neuroprotection in insulted neurons and neurotherapeutic effects in the 3xTg-AD mouse model and stimulation of AMPK/GSK3*β*(ser9) phosphorylation activity and increased expression of activated Nrf2. These results indicate that Artemether could be a new AMPK/GSK3*β*(ser9)/Nrf2 activator for the treatment of inflammation and oxidative stress and a potential therapeutic agent for Alzheimer's disease.

We found that A*β*_1-42_ caused a collapse of the *△ψ*m and increased ROS, while pretreatment with Artemether significantly suppressed these pathological changes in PC12 cells. Similarly, Artemether significantly decreased MDA levels and increased SOD activity in the 3xTg-AD mouse brain cortex. These findings propose that the antioxidant activity of Artemether is involved in its neuroprotective effects in vitro and in vivo. However, these findings are opposing the basic chemical properties of Artemether which is a prooxidant and not an antioxidant molecule. The active moiety of Artemether is an endoperoxide bridge (Figure 1(a)) that generates carbon-centered free radicals which increases oxidative stress and causes malarial protein damage via alkylation [44]. Therefore, the antioxidant effects of Artemether in the neurons and AD brain can be only explained by an indirect effect of stimulation of an endogenous antioxidant system which attempts to cope with a detrimental prooxidant neurotoxic effect induced by Artemether. The brain neurons are particularly susceptible to oxidative damage, because they contain high concentrations of readily oxidizable polyunsaturated fatty acids with high oxygen consumption rates and have only a relatively modest antioxidant defense system [45]. In order to combat oxidative stress, neurons need to activate the endogenous antioxidant defense system, such as Nrf2. Under the resting condition, Nrf2 is sequestered by Kelch-like ECH-associated protein 1 (Keap1). Upon oxidative stress, or exposure to an electrophilic agent that reacts with Keap1, Nrf2 is released from microtubule-associated Keap1 following phosphorylation of Nrf2 and nitrosylation of Keap1. Thereafter, phosphorylated Nrf2 shuttles into the nucleus, dimerizes with Maf, and binds to antioxidant response elements (AREs) in the promoter of genes encoding enzymes involved in antioxidant activities such as heme oxygenase 1 (HO-1) and SOD [46]. In the present study, we found that Artemether activated the Nrf2 ARE signaling pathway in both PC12 neuronal cultures and in 3xTg-AD mice in correlation with antioxidant and neurotherapeutic effects. There is a body of evidence that in AD patients and animal models of the AD, there is reduced Nrf2 activation and/or less shuttling to the nuclei, and that in neuronal cultures, Nrf2 activation protected towards amyloid-*β*-mediated neurotoxicity [46]. Gracilins A and C, sponge-derived diterpenoid antioxidant compounds known to activate Nrf2, were found to improve learning and spatial memory in 3xTG mice [47]. Nobiletin, a polymethoxylated flavone antioxidant isolated from citrus peels, improved cognitive impairment and reduced soluble amyloid-*β* levels in 3xTg-AD mice [48]. The present study, supported by the abovementioned cumulative findings in vitro and in AD transgenic mice in vivo, shows that Artemether, by activating the Nrf2 transcriptional pathway in redox-stressed neurons, conferred antioxidant activities which may be found useful in AD therapy.

AMPK, a heterotrimeric serine/threonine enzyme, consisting of a catalytic subunit and two regulatory subunits plays an important role as a monitoring sensor of cellular energy status [49], and its dysfunction was implicated in neurodegenerative disorders including AD [50]. The *α*-subunits are phosphorylated by upstream kinases such as Ca2+-dependent kinase CaMKK*β* (calmodulin-dependent kinase *β*) at conserved threonine residues within the activation loop (Thr172 in rat *α*1/*α*2), causing >100-fold activation. The control of AMPK activity is complex, and the classic view claims that AMPK is allosterically activated in response to intracellular stresses by the phosphorylation of the Thr172 within the *α*-subunit and/or by an increase in the intracellular AMP/ATP ratio. AMPK*α* may shuttle between the cytoplasm and the nucleus. It has also been shown that AMPK*α*1 exists predominantly in the cytoplasm, whereas the AMPK*α*2 subunit showed major localization in the nucleus [51]. Many types of stress such as oxidative stress affect the phosphorylation state of AMPK*α*1 and AMPK*α*2, causing AMPK's nuclear accumulation [52]. It is well known that AMPK phosphorylates Nrf2 at the Ser550 residue, which, together with AMPK-mediated GSK3*β* inhibition, promotes nuclear accumulation of Nrf2 for ARE-driven gene transactivation [53], enabling the cells to recover their antioxidant capacity. Present findings suggest a causal, direct relationship between Artemether-induced activation of AMPK and the neuroprotective effect towards A*β*_1_-_42_ insult and the neurotherapeutic effect in 3xTg-AD mouse model. We hypothesize that these findings are explained by Artemether-induced activation of Nrf2 resulting with antioxidant effects and neuroprotection.

Glycogen synthase kinase-3 (GSK-3) is a proline-directed serine-threonine kinase that was originally identified as a phosphorylating and an inactivating glycogen synthase [54]. Two isoforms, alpha and beta, exhibit a high degree of amino-acid homology [55]. GSK3*β* signaling plays a key role in mediating mitochondrial functions. GSK3*β* is constitutively active and is inhibited when phosphorylated at Ser9. GSK3*β* is the major kinase to phosphorylate tau proteins both in vitro and in vivo. GSK3*β* phosphorylation was associated with upregulation of antioxidant enzymes, particularly heme oxygenase-1 (HO-1) and transient elevation of intracellular glutathione (GSH) in cells exposed to acute stress [56]. Moreover, GSK3*β* phosphorylates Nrf2 to increase his proteolytic degradation [57]. It is well-established that A*β*_1-42_-induced neuronal cell death is mediated by several pathological mechanisms including GSK3*β* [58]. Here, we found that Artemether treatment increased the phosphorylation of GSK3*β* at the Ser9 site, in both A*β*_1-42_-treated PC12 neuronal cultures and in the brain cortex of 3xTg-AD mice. Therefore, these results may suggest that Artemether-induced GSK3*β* phosphorylation results to less pathological phosphorylation of tau proteins, stabilization of Nrf2 protein levels, and upregulation of antioxidant enzymes leading to neuroprotection.

Another hallmark of the AD is low-grade chronic inflammation, which is characterized by astrogliosis and microgliosis. It has been reported that Nrf2 inhibits inflammation in several neurodegenerative models by partial modulation of NF-*κ*B signaling [59]. Present results demonstrate that Artemether attenuates gliosis in correlation with upregulation of the Nrf2/HO-1 antioxidant pathway. Interestingly, it has been suggested that the anti-inflammatory activities may occur through alternative pathways independent of Nrf2. Brain glial cells produce a large number of neuroactive substances after activation, including proteases, cytokines, prostanoids, and radicals, such as nitric and oxide superoxide, which are products of the enzyme NADPH oxidase and inducible nitric oxide synthase, respectively. Glia-derived radicals, as well as their reactive reaction products peroxynitrite and hydrogen peroxide, may harm neurons and have been associated with oxidative damage and neurotoxicity in neurological diseases including AD [60]. Artemether treatment, at both low and high doses, significantly decreased glial compartment and the brain inflammatory markers IL-18 and NF-*κ*B suggesting that the neurotherapeutic effect of Artemether in the 3xTg-AD mice may be contributed by anti-inflammatory and antiglia-mediated effects, in addition to its indirect antioxidant activity. This finding is reminiscent of the anti-inflammatory beneficial effects of Artemether reported in microglia cell cultures exposed to LPS stimulation. Artemether significantly suppressed proinflammatory mediators prostaglandin E2, tumor necrosis factor-*α*, nitric oxide, and interleukin-6, through inhibition of NF-*κ*B. Artemether activated Nrf2 and increased the level of HO-1, NAD(P)H dehydrogenase [quinone] 1, and glutathione, while knock down of Nrf2 in the cells abrogated the anti-inflammatory and neuroprotective activities of Artemether [61]. These in vitro studies complement and support present findings.

## 5. Conclusion

**Figure 12:**
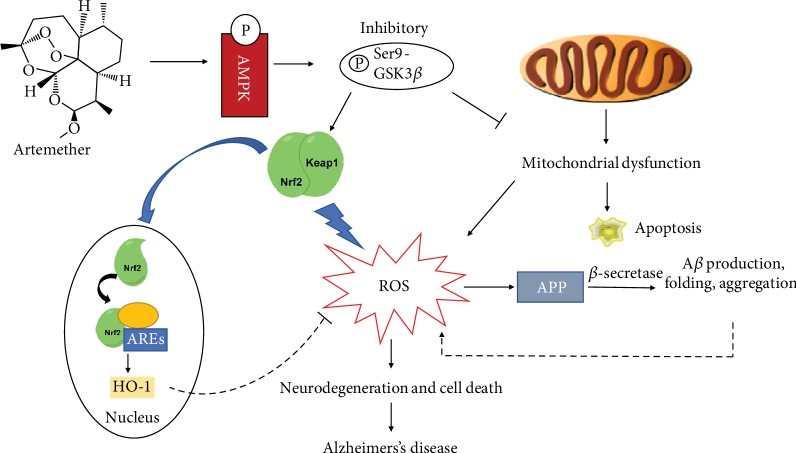
A schematic diagram of Artemether-induced AMPK/GSK3*β*(ser9)/Nrf2 activation towards reduction of amyloid-*β*-evoked oxidative stress to confer neuroprotection. Artemether stimulated AMPK/GSK3*β*(ser9) phosphorylation output in neuronal cells and animal brain cortex to increase the nuclear expression of Nrf2 with induction of the phase II antioxidant enzymes and anti-inflammatory genes containing ARE. This process (dotted line) caused attenuation of APP-derived and A*β*-induced ROS production, reduction of oxidative stress, correction of mitochondrial dysfunction, reduction of inflammation, and conferring neuroprotection.

The present study suggests that treatment with Artemether of amyloid-*β* insulted neuronal cultures conferred neuroprotection by reducing the production of ROS and correcting mitochondrial membrane potential. In 3xTg-AD mice, Artemether effectively attenuated AD symptoms (e.g., plaque proteins deposition and phosphorylation and cognitive impairment) and beneficially affected various neurodegeneration-related biochemical events linked to amyloid-*β* accumulation in the brain cortex. The most significant of these therapeutic effects were antioxidant effects, neuroprotection, and a marked reduction of neuroinflammation. At the level of signaling, Artemether modulated the phosphorylation of AMPK and GSK3*β*(ser9). These phosphorylation events most probably contributed to the correction of mitochondrial membrane potential and reduction of ROS levels. Under amyloid-*β* stressful conditions, Nrf2 can be regulated by GSK3*β*(ser9) to be released from Keap-1, shuttling to the nucleus and binding ARE resulting with antioxidant and anti-inflammatory gene activation. In turn, HO-1, SOD proteins decreased levels of ROS and anti-inflammatory mediators, decreased the inflammation process, decreased deposition of amyloid-*β* and tau hyperphosphorylation, significantly attenuated aponecrosis and other cell death processes, and finally conferred neuroprotection in vitro and in vivo in Alzheimer's disease models (Figure 12). The multimodal mechanism of action of Artemether appears to fit with the increasingly recognized multifactorial nature of AD pathology, and treatment with this potential drug can greatly benefit from its ability to act on multiple targets [62, 63]. Taken together, the results of this extensive comparative IVIVC analysis suggests that specific subset of effects not always closely related to amyloid-*β* accumulation and APP processing may play a key role in determining the neurotoxicity/neuroprotection balance. The present study proposes a new clinical drug application in AD therapy for the antimalaria drug Artemether.

## Data Availability

Data is available upon request from the author.

## Supplementary Materials

Supplementary Materials Fig. s1: Artemether-induced neuroprotective effect on A*β*_1-42_-induced cell apoptosis, mitochondrial membrane potential (*△ψ*m), and ROS in primary cultured neurons.
