# Supplementary material for: Artemether Activation of AMPK/GSK3β(ser9)/Nrf2 Signaling Confers Neuroprotection towards β-Amyloid-Induced Neurotoxicity in 3xTg Alzheimer's Mouse Model
Source: Oxid Med Cell Longev. 2019 Nov 21;2019:1862437. doi: 10.1155/2019/1862437 (PMC6907052; doi:10.1155/2019/1862437)
Supplement: Supplementary Materials — Fig. s1: Artemether-induced neuroprotective effect on Aβ1-42-induced cell apoptosis, mitochondrial membrane potential (△ψm), and ROS in primary cultured neurons. [file 1862437.f1.pdf]

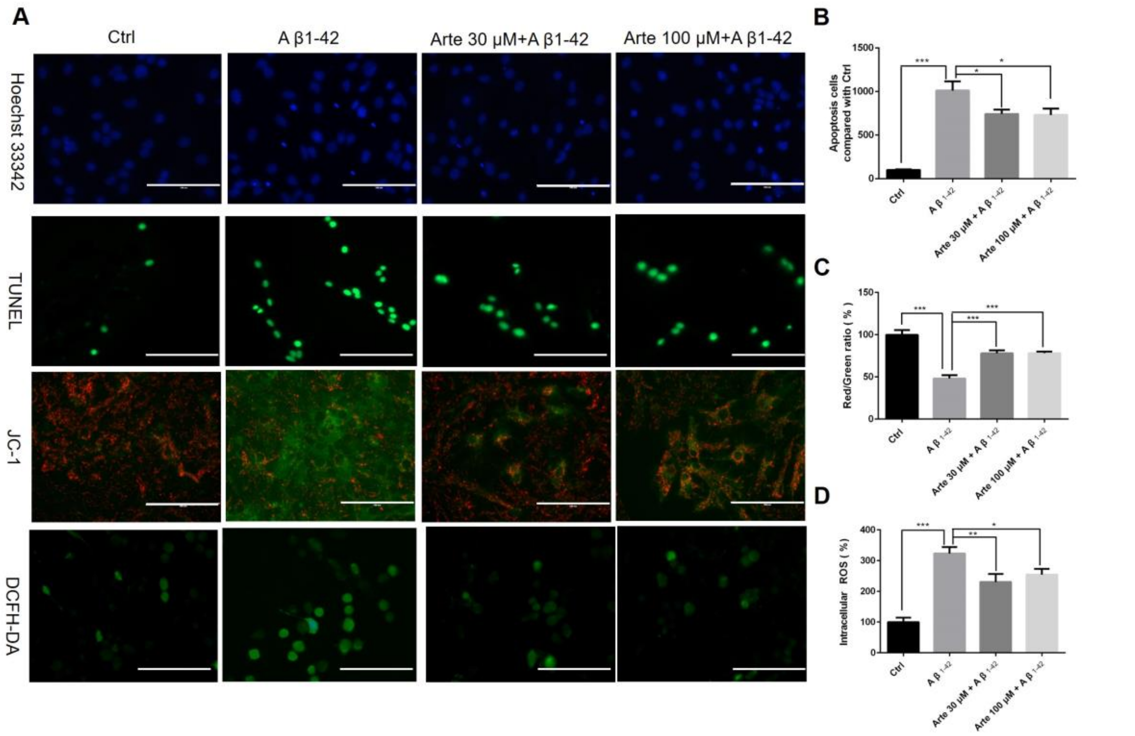


**Fig.s1. Artemether-induced neuroprotective effect on A β $_{1\text{-}42}$ induced cell apoptosis, mitochondrial membrane potential (△ψm) and ROS in primary cultured neurons.** Primary cultured neurons were pre-treated with different concentrations of Artemether for 2 h followed by exposure of 24 h to 2 μM A β $_{1\text{-}42}$. (A)Apoptotic cells were observed by Hoechst 33342 and TUNEL staining as shown in fluorescence images. And the fluorescent images represent the intracellular ROS level and mitochondrial membrane potential were determined by DCFH-DA probes and JC-1 dyes. Scale bar =100 μm. (B) Quantitation of apoptotic cell's nuclei. $^{***}$ indicates $p < 0.001$ versus the control group; $^{*}$indicates $p < 0.05$ versus the A β $_{1\text{-}42}$-treated group were considered significantly different. (C) Quantitation of the intracellular ROS level. Results are presented as mean ± SEM (n=3). $^{***}$indicates p<0.001 versus control group, $^{***}$ indicates $p < 0.001$ versus Aβ$_{1\text{-}42}$ treated group were considered significantly different. (D) Quantitation of the red to green fluorescence intensity ratio. Results are presented as mean ± SEM (n=3). $^{***}$indicates p<0.001 versus control group, $^{*}$indicates p<0.05, $^{**}$indicates p<0.01 versus Aβ$_{1\text{-}42}$ treated group were considered significantly different
